# Individual Differences in Sweetness Ratings and Cross-Modal Aroma-Taste Interactions

**DOI:** 10.3390/foods9020146

**Published:** 2020-02-01

**Authors:** Anne Sjoerup Bertelsen, Line Ahm Mielby, Niki Alexi, Derek Victor Byrne, Ulla Kidmose

**Affiliations:** Department of Food Science, Faculty of Technical Sciences, Aarhus University, 8200 Aarhus N, Denmark; annesbertelsen@food.au.dk (A.S.B.); lineh.mielby@food.au.dk (L.A.M.); niki.alexi@food.au.dk (N.A.); derekv.byrne@food.au.dk (D.V.B.)

**Keywords:** sugar reduction, sweet, vanilla, consumers, age, gender, sweet liker status, young adults

## Abstract

Aroma-taste interactions, which are believed to occur due to previous coexposure (concurrent presence of aroma and taste), have been suggested as a strategy to aid sugar reduction in food and beverages. However, coexposures might be influenced by individual differences. We therefore hypothesized that aroma-taste interactions vary across individuals. The present study investigated how individual differences (gender, age, and sweet liker status) influenced the effect of aroma on sweetness intensity among young adults. An initial screening of five aromas, all congruent with sweet taste, for their sweetness enhancing effect was carried out using descriptive analysis. Among the aromas tested, vanilla was found most promising for its sweet enhancing effects and was therefore tested across three sucrose concentrations by 129 young adults. Among the subjects tested, females were found to be more susceptible to the sweetness enhancing effect of vanilla aroma than males. For males, the addition of vanilla aroma increased the sweet taste ratings significantly for the 22–25-year-olds, but not the 19–21-year-olds. Consumers were clustered according to their sweet liker status based on their liking for the samples. Although sweet taste ratings were found to vary with the sweet liker status, aroma enhanced the sweetness ratings similarly across clusters. These results call for more targeted product development in order to aid sugar reduction.

## 1. Introduction

Excessive sugar intake has contributed to the prevalence of obesity and associated lifestyle diseases, such as type 2 diabetes [1,2]. Reduction of the content of sugar in food and beverages is therefore of high importance for both society and industry. Previously, non-nutritive sweeteners have been used extensively for sugar reduction purposes in the food and beverage industries [3]. However, many non-nutritive sweeteners have a negative effect on consumers’ acceptability of the products, as they can have off-flavors and a slow onset or a lingering of the sweet taste [3,4]. As eating and drinking are multisensory experiences, cross-modal interactions have been suggested as an alternative strategy in the reformulation of products with the aim of reducing the sugar content [5,6,7,8]. The addition of aroma has, for example, been shown to increase sweetness intensity in many studies [5,9,10,11,12,13,14,15] and has therefore been suggested as a tool to aid sugar reduction. However, the effect of aroma on sweetness intensity is dependent on several factors, such as tastant concentration [14,16,17,18,19], aroma concentration [5,14,16,20,21], and the food matrix [5,7]. In addition, the type of aroma used has also been found to affect the ability to enhance sweetness intensity [5,9,13,15,22,23]. A careful screening of aromas is therefore of high importance in the development of sugar reduced products. 

The extent to which an aroma is able to enhance sweetness intensity has been suggested to depend on whether the pairing of stimuli is congruent or not. Schifferstein and Verlegh [16] defined congruency as “the extent to which two stimuli are appropriate for combination in a food product”. While some authors have found that congruency is necessary for aromas to enhance sweetness intensity [16,17,22], Wang et al. [15] found that both a congruent (vanilla) and an incongruent (beef) aroma could enhance sweetness intensity in sweetened milk. However, the sweetness enhancement effect was shown to be higher for the congruent compared to the incongruent aroma [15]. The majority of aromas that have been found to enhance sweet taste intensity are thus congruent with food products high in sweetness intensity, such as fruity or vanilla aromas. Sweetness enhancement by aroma is accordingly associated previous coexposures whereby specific combinations of aromas with sweet taste are learned [21,24,25].

Individual differences, such as age or gender, might possibly affect the sweetness enhancement ability of aromas due to either physiological differences or differences in consumption behavior between consumer groups. Doty and Cameron [26] suggested that for at least some aromas, females are more sensitive than males regarding odor detection, identification, discrimination, and memory. Other studies suggest that sweetness sensitivity decreases with age [27,28,29]. Differences in sensitivity might possibly change the perception and preference for food products. Indeed, older consumers with lower sensitivity for various stimuli were found to prefer higher intensity of sweetness than consumers with higher sensitivity [30]. Males and females have also been found to differ when it comes to their preferences for sweet taste and related products [31,32]. This might lead to differences in consumption behavior. Indeed, individuals with a higher preference for sweet taste have been found to have a higher intake of sweet beverages than individuals with a lower preference for sweet taste [33,34]. Different consumption behaviors might affect coexposures, and as explained previously, therefore also affect the abilities of aromas to enhance sweetness intensity. However, few studies have actually investigated the effect of individual differences on cross-modal interactions. Proserpio et al. [35] recently investigated how BMI, gender, and butter aroma in two different concentrations affected sweet taste intensity in custard desserts. For obese individuals, they found that females experienced a cross-modal effect of aroma on sweetness intensity for both aroma concentrations tested, while males only experienced this for the highest aroma concentration tested. However, for the normal weight individuals, no differences in ratings of sweetness intensity between genders were observed. Therefore, for obese individuals, females appeared to be more susceptible to the cross-modal effect than males. Moreover, Philipsen et al. [36] investigated the effect of age and aroma on sweetness intensity. They did not find a difference in the effect of cherry flavor on sweetness intensity between the group aged 18–22 years versus the group aged 60–75 years. Lavin and Lawless [37] similarly investigated the effect of age and vanilla flavor on sweet taste intensity in milk, but among different age groups: 5–7, 8–10, 11–14, and 18–31-year-olds. The only significant difference between groups in relation to sweetness enhancement of vanilla flavor was a less pronounced enhancement in sweetness intensity for the 5–7-year-olds in comparison with the 8–10-year-olds. However, as mentioned above, cross-modal aroma-sweetness interactions depend on many factors such as the type of aroma, their concentrations, and the food matrix. As these factors might also interact with the individual differences, the results of the mentioned studies might therefore not be valid for aromas, concentrations, and matrices different from those tested in each study. To the authors’ knowledge, no studies have investigated the effect of sweet liker status on the cross-modal effect of aroma on sweetness intensity. Further studies are therefore warranted.

In the present study, we hypothesized that individual differences can affect the cross-modal effect of aroma on sweetness intensity. The aim was therefore to investigate how individual differences (gender, age, and sweet liker status) influence the effect of a congruent aroma on the dependent variable sweetness intensity. Besides ratings of sweetness intensity, the intensity of other sensory attributes as well as liking were also measured in order to better understand the extent of smell-taste cross-modal interactions and to be able to categorize individuals according to sweet liker status. As particularly adolescents and young adults have been found to consume too much sugar and sugar sweetened beverages [38,39,40,41], the consumer group used for this study consisted of young adults.

## 2. Experimental Design

**Figure 1:**
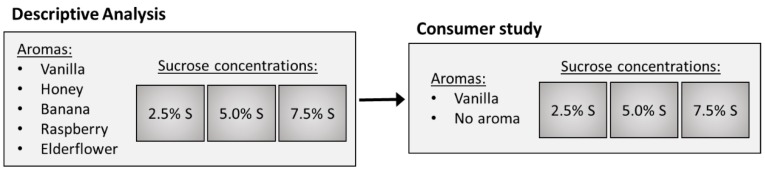
Overview of the samples in the descriptive analysis and the consumer study. % S = % *w/w* sucrose.

In order to select an aroma for the consumer study, the first experiment was a screening of five aromas (vanilla, honey, banana, raspberry, and elderflower) which were all congruent with sweetness in a Danish cultural context. As descriptive sensory analysis (DA) previously has shown to be a successful method to identify aroma-related sensory interactions [42], DA was used for the screening of aromas. Based on the results of the DA, vanilla aroma was chosen for the consumer study (Experiment 2). Figure 1 provides an overview of the samples included in the DA as well as in the consumer study.

### 2.1. Samples

Fifteen samples were evaluated in the DA, while six samples were evaluated in the consumer study. Samples were prepared according to Table 1. All aromas were purchased from Bolsjehuset (Albertslund, Denmark) and the aroma concentrations (Table 1) were selected based on the recommendations from the supplier. As described in the introduction, the effect of aroma on sweetness intensity is dependent on the tastant concentration. The aromas were therefore combined with three different sucrose concentrations: low sucrose concentration (2.5% *w/w*), medium sucrose concentration (5% *w/w*), and high sucrose concentration (7.5% *w/w*) in aqueous blends based on local tap water. Sucrose was purchased from Merck KGaA (Darmstadt, DE) and due to the aim of sugar reduction, the sucrose concentrations were chosen to be lower than the 10% sugar, which is normally present in many beverages [43]. The sucrose concentrations were additionally pilot tested to give perceivable differences in sweetness intensity.

Samples were prepared by first mixing sucrose with water. When the sucrose was dissolved, aroma was added according to Table 1. Samples were then poured into opaque sample tubes with red lids (Fisher Scientific, Roskilde, Denmark) with 20 mL in each. Prior to evaluation, samples were stored in 5 °C for 1–3 days. In both the DA and the consumer study, samples were coded with three-digit numbers and served at room temperature.

## 3. Experiment 1—Screening of Aromas

The objective of the first experiment was to screen aromas for their enhancing effect on sweetness intensity, in order to select the most effective one for the consumer study.

### 3.1. Method

The DA was done by an experienced trained sensory panel in the sensory lab at the Department of Food Science, Aarhus University, Årslev, Denmark. The panel consisted of nine women, with an age range of 22–60 years, who all gave informed verbal consent prior to participation. The DA began with an introductory discussion, followed by training and the final evaluation of the samples. In the introductory discussion, samples that were expected to span the relevant attributes were presented and discussed with the panel. During the discussion, the panel chose to continue with the following attributes (evaluated in the order shown): sweet aroma, sour aroma, intensity of aroma, sweet taste, sour taste, and intensity of flavor, as these attributes were able to describe the sensory differences within the samples presented. To allow for detection of differences in aroma and flavor attribute intensities, control samples without aroma were not included in the DA. In the training session, panelists evaluated a subgroup of the samples in triplicate.

The samples were evaluated in triplicates during the final evaluation, which took place over two consecutive days. For each panelist, samples were presented according to a Williams Latin Square design to limit carry over effects [44]. Attribute intensities were evaluated on 15 cm line scales with two anchor points placed at 1.5 and 13.5 cm of the scale, indicating low and high intensity, respectively. Data was recorded directly in the software Compusense (Compusense Inc., Ontario, Canada). A 30-s break was enforced between samples during which panelists were instructed to cleanse their palate using water, tepid very weak white tea, and/or crackers.

### 3.2. Statistical Analyses

For the DA, analysis of variance (ANOVA) and Tukey’s HSD multiple comparison tests with *p* ≤ 0.05 as significance level were performed for each attribute. This was done using XLSTAT (2018.6) (Addinsoft, NY, USA) [45]. Sucrose, aroma, and their interaction were treated as fixed effects, while panelists, replicates, and interactions were treated as random effects. None of the three-way interactions were significant and all models were therefore reduced to only include two-way interactions. To compare differences in sweetness intensity between aromas, ANOVAs were run with sucrose concentration as a fixed effect and panelists as a random effect. In order to visualize how the attributes and samples were correlated, Principal Component Analysis (PCA) of the mean centered average results from the DA was also performed with XLSTAT.

### 3.3. Results

A significant interaction between aroma and sucrose was found for the attributes sweet (F_(8,304)_ = 2.02, *p* = 0.044) and sour taste (F_(8,304)_ = 2.71, *p* = 0.007) (Table 2). This demonstrated that the effects of aroma and sucrose were interdependent for the rating of both sweet and sour taste intensity. Different cross-modal interactions were thus identified: aroma affected ratings of taste intensity, and sucrose affected the rating of retro-nasal aroma intensity (flavor intensity (F_(2,304)_ = 17.52, *p* = < 0.0001)). Samples with added vanilla aroma were in all sucrose concentrations rated the most sweet, while samples with added elderflower aroma were generally rated as the least sweet. Sucrose concentration significantly affected the enhancing effect of vanilla aroma compared to elderflower aroma on ratings of sweet taste intensity (F_(2,16)_ = 6.17, *p* = 0.010). The difference in sweet taste scores was significantly lower for the high sucrose concentration (difference = 4.5) than for the medium (difference = 6.6, *p* = 0.011) and low sucrose concentrations (difference = 6.2, *p* = 0.047). These results indicate generally higher cross-modal effects in the medium and low sucrose concentrations when compared to the high sucrose concentration.

**Figure 2:**
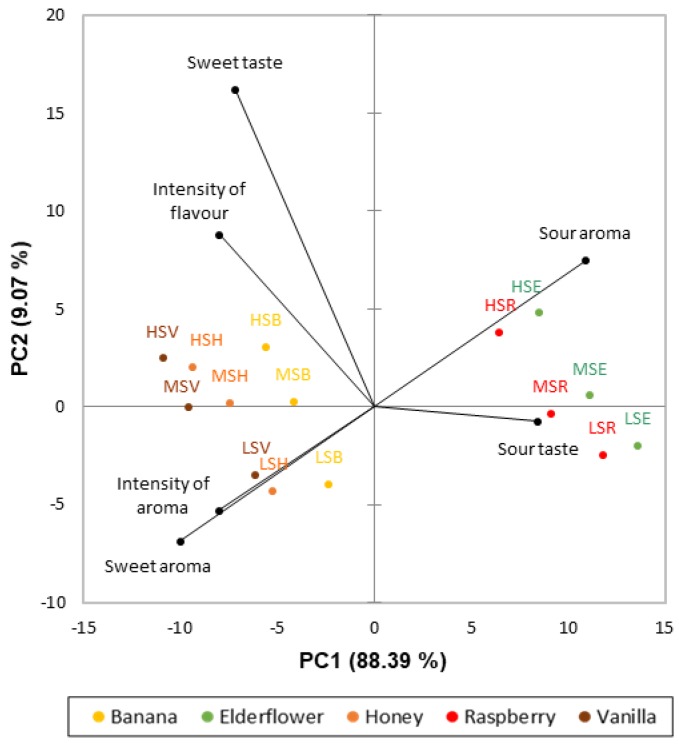
Principal Component Analysis biplot of the results from the DA. Attributes are marked in black while samples are colored according to aromas and coded according to sucrose concentration: LS = low sucrose concentration, MS = medium sucrose concentration, HS = high sucrose concentration, and aroma type: B = banana aroma, E = elderflower aroma, H = honey aroma, R = raspberry aroma, and V = vanilla aroma.

Figure 2 shows a PCA biplot of the results from the DA. In total, the first two Principle Components (PCs) explain 97.46% of the variance in the data. As shown, the attribute sweet aroma was negatively correlated to the attribute sour aroma (Pearson’s correlation = −0.81, *p* < 0.0001); thereby creating a sweet-sour aroma dimension, that separated the samples into two groups. Samples with added vanilla, honey, and banana aromas formed one group that was characterized by an intense sweet taste, sweet aroma, high overall intensity of aroma and flavor. The other group was formed by the samples with added raspberry or elderflower aromas and was characterized by intense sour aroma and taste.

For each sucrose concentration, samples with added vanilla aroma were rated as having the highest intensities of sweet aroma (significant result, *p* < 0.01) and sweet taste (non-significant result, *p* = 0.410–1.000). Vanilla aroma was therefore found to be most promising in relation to sweet taste enhancement from a cross-modal effectiveness perspective. To follow up, the consumer study was set up to test the cross-modal effect of vanilla aroma across the same sucrose concentrations.

## 4. Experiment 2—Effect of Individual Differences in Consumers

The aim of the second experiment was to investigate the effect of individual differences (gender, age, and sweet liker status) on the enhancing effect of vanilla aroma on ratings of sweetness intensity. In order to better understand the extent of the aroma-taste interactions, results will also be presented for the attribute sweet aroma intensity.

### 4.1. Method

In order to recruit subjects from the relevant target group of young adults, the study was conducted in two different canteens at Aarhus University, Aarhus, Denmark. The study was carried out over two consecutive days and in total, 129 young adults (mean age 21.9 ± 1.5 years, 51 males) participated. After the subjects had agreed to participate, they were served the six samples together with a paper questionnaire, a pen, and water for rinsing the palate between the samples. They were then given a short verbal introduction to the assignment and asked to match the three-digit numbers in the questionnaire with the three-digit numbers on the samples. For each sample, they rated the intensities of sweet aroma, sweet taste, and sour taste, as well as overall liking on nine-point scales anchored with “not at all” and “extremely” corresponding to point 1 and 9 on the scales, respectively. The liking scale also included an anchor in the middle named “neutral” corresponding to 5. To control for presentation bias, different versions of the questionnaires that differed in presentation orders were prepared and used. After participation, all subjects were offered small treats as compensation for their time. Prior to the actual consumer study, the procedure and questionnaire were pilot tested.

### 4.2. Statistical Analyses

For the consumer study, consumers were divided into two age groups: 19–21-year-olds (*n* = 56, 20 males) and 22–25-year-olds (*n* = 73, 31 males). These groups were chosen as they gave the most equal and balanced groups in relation to total numbers of subjects as well as between genders. Using Minitab®19 (19.1.1) (Minitab, LLC, State College, PA, USA), mixed-model ANOVA and Tukey’s HSD multiple comparison tests with *p* ≤ 0.05 as the significance level, were carried out for the sensory attributes sweet aroma and sweet taste. Sucrose, aroma, age, gender, and their interactions were considered to be fixed effects, while location (canteen), subject nested within age and gender, and interactions involving these were treated as random effects. Location was found not to have any significant main or interaction effect. Thus, location as a factor was excluded from the reduced models. Gender was found to have a significant effect on several of the attributes. To examine the effect of gender, the dataset was thereafter split into two datasets: a “male” and a “female” dataset. The same ANOVA models as described above (excluding the gender factor) were then performed on the occurring datasets. As the three-way interaction between sugar, aroma, and age was not significant in any of the models, only two-way interactions were included in the final reduced models.

To identify groups of subjects with similar preferences, Agglomerative Hierarchical Clustering (AHC) analysis was performed on the liking data in XLSTAT by applying the Euclidean distances and Ward’s agglomeration method. The clusters were compared in terms of gender and age group distribution using chi-square tests. Thereafter, ANOVA models were again carried out with sucrose, aroma, cluster, and their interactions as fixed effects, while subject nested in cluster was treated as a random effect, using the Minitab®19 software.

### 4.3. Results

#### 4.3.1. Effect of Gender and Age among Young Adults on Ratings of Sweet Aroma and Sweet Taste Intensity

In Table 3, the results of the consumer study are shown separately for males and females. The addition of vanilla aroma increased the rating of sweet aroma intensity significantly for both genders (males: F_(1,49)_ = 461.42, *p* < 0.001; females: F_(1,76)_ = 866.52, *p* < 0.001), but for males, a significant interaction effect between sucrose and aroma was also identified (F_(2,100)_ = 4.82, *p* = 0.010). For the samples with added vanilla aroma, the sample with the medium sucrose concentration was rated significantly higher in sweet aroma intensity than the sample with the high sucrose concentration (*p* = 0.001). The sweet aroma intensity of the sample with low sucrose concentration was rated lower than the sample with medium sucrose concentration and higher than the sample with the high sucrose concentration, neither however significantly (*p* = 0.185 and *p* = 0.433, respectively).

A significant sucrose-aroma interaction for sweet taste intensity was found for both genders (males: F_(2,100)_ = 7.22, *p* = 0.001; females: F_(2,154)_ = 24.59, *p* < 0.001). For males, just one significant cross-modal effect of vanilla aroma on sweet taste intensity was found, which was an enhancement of sweetness for the low sucrose concentration (*p* < 0.001). For females, a significant cross-modal effect of vanilla aroma on sweet taste intensity was found at both the low (*p* < 0.001) and high (*p* < 0.001) sucrose concentrations.

For males, a significant interaction effect was also seen between age and aroma affecting sweet taste intensity (F_(1,49)_ = 5.69, *p* = 0.021). The younger group of males rated the samples equally sweet independent of whether the samples were added vanilla aroma or not (*p* = 0.972). However, the older group of males rated the samples with added aroma significantly sweeter (mean 6.2) than the samples without aroma (mean 5.1) (*p* < 0.001). The cross-modal effect of vanilla aroma on sweet taste intensity thereby not only seems to be dependent on gender, but also on age for young males. For females, a significant main effect of age was seen on the rating of sweet taste intensity (F_(1,76)_ = 7.22, *p* = 0.009). For 19–21-year-old females, the mean sweet taste intensity score of all samples was 5.9, while the mean of the sweet taste intensity score for the 22–25-year-old females was 6.5. The older group of females thereby rated the samples sweeter than the younger group.

#### 4.3.2. Consumers’ Acceptance and its Effect on Ratings of Sweet Aroma and Sweet Taste Intensity

For the consumer study, the subjects were divided into three clusters based on their liking of the samples. In general, samples acquired liking below the neutral point (grand mean ± SD = 4.2 ± 2) of the nine point scale. However, this was expected since the samples were aqueous blends and therefore very different from beverages normally consumed. In Table 4, gender and age characteristics for each of the clusters are shown together with the liking centroids.

When looking at the liking centroids for the clusters (Table 4), it seems the clusters can be characterized according to their preferences for sweetness intensity. The liking for the samples in Cluster 1 seems to increase along with the increase in sucrose concentration. No relation between either sucrose concentration or vanilla aroma and liking seems to exist for Cluster 2. For Cluster 3, liking seems to decrease with increasing sucrose concentration and the addition of vanilla aroma. According to this, the clusters can be described as sweet likers (1), neutrals (2), and sweet dislikers (3). No significant differences were found in the distribution of age groups between clusters (*p* = 0.478). The gender distribution was however skewed (*p* < 0.0001). Relative to the total number of males in the consumer study, there was a predominance of males among both the sweet likers and the neutrals. In the group of sweet dislikers, there was a predominance of females. To study if there was an effect of the clusters on the subjects’ ratings of the sensory attributes, ANOVAs were performed with cluster included in the models as a factor (Table 5).

**Figure 3:**
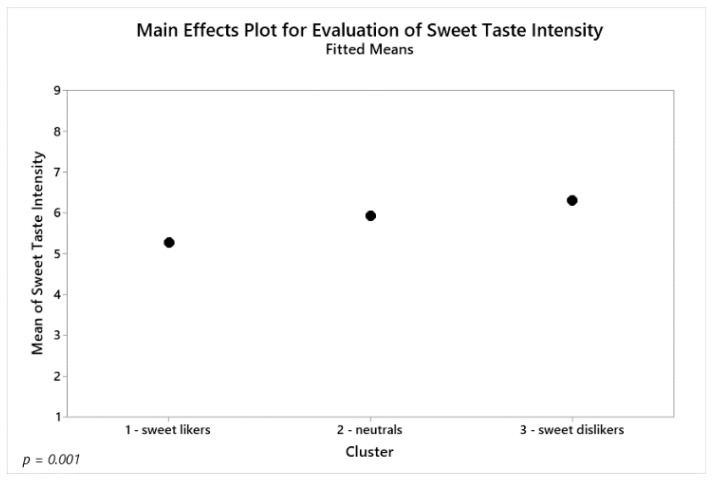
Fitted means of rated sweet taste intensity for the three clusters found in the consumer study.

In accordance with the previous ANOVAs (Table 3), addition of vanilla aroma was found to increase the rating of sweet aroma intensity significantly (F_(1,126)_ = 53.97, *p* < 0.001). Furthermore, an interaction between sucrose and aroma significantly affected the rating of sweetness intensity (F_(2,256)_ = 30.55, *p* < 0.001). The addition of vanilla aroma only increased sweet taste intensity at the low and high sucrose concentrations. This is similar to the results previously described for females in Section 4.3.1. Besides sucrose and aroma affecting sweetness intensity, the clusters were also found to significantly vary in their ratings of the sweetness intensity (F_(2,126)_ = 7.67, *p* = 0.001). As illustrated in Figure 3, sweet likers generally rated the sweet taste intensity of the samples significantly lower (mean 5.3) than both the neutrals (mean 5.9, *p* = 0.045) and the sweet dislikers (mean 6.3, *p* < 0.001) did. The neutrals and the sweet dislikers did not rate the sweet taste intensity significantly differently (*p* = 0.123).

## 5. Discussion

### 5.1. Screening of Aromas

The DA revealed a sweet-sour aroma dimension (Figure 2), which is in accordance with Chifala and Polzella [46], who found a sweet-sour dimension when analyzing orthonasal evaluations of liqueurs using multidimensional scaling. The sweet-sour dimension in the present study was found to divide the aromas into two distinct perceptual groups: a “sweet” and a “sour” group. Aromas have previously been described with taste-like qualities such as sweet or sour even though they do not elicit tastes per se. This distinction of aromas being perceived either mainly as sweet or sour is likely created due to previous coexposures with sweet or sour taste qualities whereby a cognitive association of the aromas with the taste qualities is produced. Thus, aromas paired with either sucrose or citric acid have previously been found to subsequently smell sweeter and more sour than before pairing, respectively [47,48]. Raspberry and elderflower aromas therefore seem to be more associated with sour, rather than sweet taste. The aroma-induced sour taste of raspberry and elderflower samples, as seen in the DA, might therefore possibly have suppressed the sweet taste of these samples, as sour taste is known to suppress sweet taste due to taste-taste interactions [49]. The fact that the samples with added aromas from the “sweet” aroma group were consistently rated significantly higher in sweetness intensity than the samples with added aromas from the “sour” group is in accordance with the sweetness enhancing effect of aromas being dependent on how “sweet” they smell. Indeed, how “sweet” aromas smell has been shown to predict 60% of the sweet taste enhancement by aromas [21]. However, Barba et al. [23] found that just two out of nine aroma compounds associated with sweetness significantly increased the sweet taste intensity of 7% sucrose in water.

Among the aromas tested, the addition of vanilla aroma, which belonged to the “sweet” aroma group, led to the highest intensities for both sweet aroma and sweet taste (Table 2). Moreover, vanilla aroma has previously shown to increase sweetness intensity in several studies [5,7,14,15,18,37,50,51] and was thus found the most promising aroma with respect to sweet taste enhancement.

### 5.2. Effect of Gender on the Cross-Modal Effect of Aroma on Sweet Taste Intensity

In the consumer study, females seemed to be more susceptible for the cross-modal effect of vanilla aroma on sweetness intensity compared to males as the addition of vanilla aroma significantly increased sweet taste intensity scores at two sucrose concentrations for females, but just at one for males. To our knowledge, just one study has previously investigated the effect of gender on cross-modal aroma-sweetness interactions. Proserpio et al. [35] investigated the effect of BMI and gender on the evaluation of custard desserts added either no, 0.05%, or 0.1% butter aroma. They found a significant interaction effect between gender, BMI, and samples on the rating of sweet taste intensity. For obese subjects, females seemed to be most susceptible to the cross-modal effect of butter aroma on sweet taste intensity, while there for normal weight subjects were no differences between genders. In contrast, BMI was not investigated in the present study, and the current results can therefore not be directly compared with the study by Proserpio et al. [35].

Although conflicting results exist, most studies, according to Doty and Cameron’s review on the subject, suggest that females on average are more sensitive than males when it comes to their ability to smell [26]. This might possibly explain why females in this study are more susceptible towards the cross-modal effect of vanilla aroma on sweetness intensity. A higher sensitivity might lead to females perceiving aromas as either stronger or more often than males. This in turn might cause females to experience coexposures of aromas with sweet taste either stronger or more frequently, and thus facilitating associative learning. Likewise, females perform better when it comes to odor memory [26]. Therefore, the gender differences seen in this study might also possibly be due to psychological or cognitive differences between males and females. For example, perhaps females are better at matching or remembering learned associations of tastes and aromas.

### 5.3. Effect of age on Ratings of Sweet Aroma and Sweet Taste Intensity

In the consumer study, a significant interaction between age and aroma was found affecting sweet taste intensity for males. For the younger group of males, no cross-modal effect of aroma was seen, while the older group of males rated samples with added vanilla aroma sweeter than samples without added aroma. Differences in cross-modal effects with age might possibly be due to differences in products normally consumed by each age group resulting in different associative learning. In 2017, 65.8% of the new students in the Danish universities were 21 years or younger [52]. As the consumer study was performed in the university canteens, it is therefore reasonable to assume that the majority of consumers in our study, especially in the younger group, were in the beginning of their university studies. Changes in consumption behavior with age in this group of consumers could thus occur due to changes in the students’ life such as new friends or new living arrangements. As for the effect of gender on cross-modal aroma-sweetness interactions, just a few studies have investigated the effect of age on these interactions. The focus of these studies has primarily been on young adults versus senior adults (>60 years old) [30,36], and do therefore not compare relatively small age differences as seen in this study. One study however investigated the effect of adding vanilla flavor to milk among three different age groups of American children: 5–7, 8–10, and 11–14-year-olds, as well as for young adults aged 18–31 years old. All age groups found the sample with added vanilla flavor sweeter than the sample without. The only difference found was between the 5–7-year-olds and the 8–10-year-olds as the sweetness enhancement from vanilla flavor in the younger group was less pronounced than in the older group [37].

In this study, age did not affect the cross-modal effects for females, but it did have an effect on sweet taste intensity as the older group of females generally rated samples to be sweeter than the younger group of females. In contrast to cross-modal effects across ages, several studies have investigated taste perception across the life span. While some studies have found that neither the rating of sweet taste intensity nor the detection threshold of sucrose were affected by age in adults [53,54], others have found a decrease in sweetness sensitivity with age, especially after the late fifties [27,28,29]. As for the cross-modal interactions, few studies have focused on the effect of age in younger adults. Yamauchi et al. [55] studied different age groups of a Japanese population and found an interaction between age and gender on the detection threshold for sweet taste. Age (15–17 vs. 20–29- year-olds) affected sweet taste detection for men, but not for women. However, in the present study, supra-threshold intensities, and not detection thresholds were investigated, and these two parameters might not be correlated. Indeed, Mojet et al. [56] found that within 21 younger subjects between 19 and 33 years old, there was no relationship between threshold sensitivity and supra-threshold intensity in water or food products. Threshold sensitivity might therefore not be suitable to predict sensitivity of supra-threshold intensities in this group of consumers. Most recently, Dinnella et al. [57] among other things investigated the effect of individual differences on rated sweetness intensity of an aqueous 20% w/w sucrose solution among 1119 subjects. They found that neither fungiform papillae density, gender, nor age class (18–30, 31–45, and 46–60-year-olds) significantly affected the rated sweetness intensity of the aqueous solution in either 6-*n*-propylthiouracil (PROP) non-tasters or super-tasters. Besides differences in the age classes investigated, the different result from this study might also be due to the different sucrose concentrations used. Indeed, Dinnella et al. [57] wrote that intensity of sweetness was designed to be moderate/strong on a general Labeled Magnitude Scale, while the sucrose concentrations in this study were chosen to be lower than the sugar concentration normally found in beverages (10%) [43]. Finally, the discrepancy between the study of Dinnella et al. and our study might also be due to the population size, since our population size was much smaller than the population investigated by Dinnella et al. [57].

### 5.4. Effect of Sucrose on Release of Aroma

In the consumer study, samples with added vanilla aroma were rated significantly higher in sweet aroma intensity compared to samples without added aroma. Interestingly though, an interaction between sucrose and aroma was found affecting the attribute sweet aroma intensity for males. The change in the aroma intensity noted between sucrose levels might be due to a chemical interaction between the sucrose molecules and the aroma compounds in the solution, changing the release of the aroma compounds. Indeed, the addition of sucrose (20% *w/w*) has previously shown to change the release of various aroma compounds compared to pure water [58]. In this way, sucrose seems to inhibit the release of the vanilla aroma. However, the sample with the low sucrose concentration was evaluated to be between the samples with medium and high sucrose concentrations in relation to the rating of sweet aroma intensity. The order of the samples with added vanilla aroma does not therefore follow the sucrose levels according to the rating of sweet aroma intensity, indicating that this might also be interpreted as a random effect. Another aspect supporting this view is that the sucrose-aroma interaction was only seen for males and not females. This might be explained by the fact that males are more sensitive to the aroma than women, but as mentioned earlier the opposite has been found more often [26]. This sucrose-aroma interaction affecting the rating of sweet aroma intensity for males does therefore not seem likely, but cannot be ruled out.

### 5.5. Classification of Sweet Liker Status and the Effect on Ratings of Sweet Taste Intensity

The description of the clusters in the consumer study as sweet likers, neutrals, and sweet dislikers is in accordance with previous classifications of consumers according to their preferences for sweet taste [34,59]. In this study, 16.3% of consumers were classified as sweet likers, 42.6% as neutrals, and 41.1% as sweet dislikers. Iatridi et al. [59] reviewed different classification methods with aqueous sucrose solutions to determine sweet liker status and calculated the total weighed average proportions of the different sweet taste liker phenotypes. Across methodologies they found 48.5% sweet likers, 48.2% sweet dislikers, and 3.3% others/undefined phenotype. The biggest difference from Iatridi et al.’s results, therefore, seems to be a smaller proportion classified as sweet likers and a bigger proportion classified as neutrals in this study. Iatridi et al. [59] identified five overall classification methods used to determine sweet liker status: “Visual discrimination of hedonic responses”, “Statistical discrimination of hedonic responses (algorithmic classification)”, “Highest preference using ratings”, “Average liking above mid-point”/”positive–negative average liking”, and “Highest preference via paired comparisons”. Compared to the other classification methods identified, the “Statistical discrimination of hedonic responses (algorithmic classification)”, such as for example AHC analysis as used in this study, generally classified a higher proportion of the unclassified phenotypes [59]. Yet, even when accounting for this, the proportional difference of neutrals/unclassified phenotypes between the two studies is still big. The differences in distribution might therefore result from individual preferences for the vanilla aroma as well as a smaller range of sucrose concentrations tested in this study.

The distribution of the two age groups in the three clusters was not skewed, but the distribution of genders was. For the sweet likers and the group of neutrals, there was a predominance of males compared to the total number of males in the consumer study. For the sweet dislikers, there was instead a predominance of females. The skewed gender distribution for sweet dislikers (relatively more females than males) is in accordance with Yeomans et al. [31] who found that males consistently rated liking for sweetness higher than females. Similarly, Deglaire et al. [32] found that males rated liking for sweet foods and sugar higher than females across different BMI categories. Tuorila et al. [60] also found gender differences in the responses to sweetness. However, these referred to differences dependent on nationality and age, whereas others have found that there were no significant associations between gender and sweet liker/disliker status [4,61]. According to Deglaire et al. [32] a difference between males and females in their preference for sweet taste might possibly be due to different levels of health consciousness, as females have been shown to have higher health-related nutrition knowledge [62] and to find healthy eating more important than males [63,64,65].

As mentioned, ANOVAs were performed to evaluate whether the different clusters rated the samples similarly. The results showed that the clusters rated the sweet taste intensity differently from each other. Previous studies on the association between sweet liker status and sweetness intensity ratings are conflicting. As in this study, some studies have found that sweet dislikers generally rate sweetness intensity higher than sweet likers [61,66,67]. This might indicate that sweet dislikers perceive the same sucrose concentrations as more intense than sweet likers, and therefore do not like them. If this is actually the case, where the differences are due to differences in perception rather than hedonic preferences, then the sweet liker/disliker status might be an incorrect wording of the classification. However, others have found that sweet liker status is not associated with differences in ratings of sweetness intensity [4,68,69]. Differences might possibly be due to different methods used. Indeed, both Methven et al. [67] and Yang et al. [61] found their results to be dependent on matrix. Methven et al. [67] found that sweet dislikers rated sweetness intensity higher than sweet likers in jelly, but not in orange juice, while Yang et al. [61] found that low sweet likers (sweet dislikers) generally rated sweetness intensity of sucrose solutions higher than other groups, while this was not the case in iced tea.

### 5.6. Limitations and Future Research

When analyzing the effects of individual differences such as age, gender, and sweet liker status, as done in this paper, it is important to keep the size of the population investigated in mind. The population sizes became relatively small when the dataset was split into both age and genders in the present study. The results seen, especially for the interaction between age and gender, could thus be random. Future studies should consequently aim to confirm our results with a larger population. It would also be interesting to reveal whether these results can be generalized to other populations such as other cultures, or other age groups regarding the gender effect.

As gender differences were found for the cross-modal effect of vanilla aroma on sweet taste intensity in the consumer study, it would have been preferable to have had a gender-balanced panel for the DA. Nevertheless, women were chosen for the panel because they performed better during the recruitment and annual retests of panelists. In the future, however, the results on individual differences from the present study should be considered when recruiting trained panelists for work on cross-modal interactions.

Another important factor to keep in mind is the food matrix. As described previously, the matrix for example affects whether sweet dislikers rate sweetness intensity higher than sweet likers or not [61,67]. In the present study, water was used as a model system. Additional studies with other more complex food matrices should be conducted in order to reveal if similar results can be obtained as in this study. On average, the samples in our study were not very liked. This could possibly be because water is not congruent with sweet taste and/or vanilla aroma. Indeed, congruency has been found to affect pleasantness of aroma-taste mixtures [16,70]. As aroma-taste interactions have also been suggested to be dependent on the matrix/product as well as previous food experience [5], future studies should investigate how congruency with the matrix affects aroma-taste interactions. Finally, it should be investigated whether the results also apply to other aromas than vanilla aroma.

## 6. Conclusions

To study individual differences in cross-modal effects among young adults, five aromas were first screened for their sweetness enhancing effect across three sucrose concentrations. Interestingly, the five aromas, all congruent with sweet taste, were found to belong to two distinct perceptual groups: a “sweet” and a “sour” group. Vanilla aroma belonged to the “sweet” group and was evaluated as the most promising aroma in relation to sweetness enhancement. Based on these results vanilla aroma was used for the consumer study that followed. Among young adult consumers, females were found more susceptible to the sweetness enhancing effect of vanilla aroma than males were. Furthermore, different age effects were found for males and females. For males, a significant cross-modal effect of vanilla aroma on sweet taste intensity was only found for the 22–25-year-olds, but not the 19–21-year-olds. While for females, the 22–25-year-olds were generally found to rate samples sweeter than the 19–21-year-olds. Finally, subjects were separated according to their sweet liker status. Interestingly, the groups identified were found to differ in their ratings of sweet taste intensity, but not in cross-modal effects. Sweet likers, a cluster with a predominance of men, rated sweet taste intensity significantly lower than the sweet dislikers, which were predominantly women. These results indicate that, even within a relatively small target group of young adults, individual differences in perception and in cross-modal interactions exist. These results could be used for more targeted product development and marketing in order to successfully aid sugar reduction and improve healthy eating. Furthermore, these results contribute to our understanding of cross-modal interactions and sensory integration. However, as results are restricted to the aroma and matrix tested, it would be interesting to further investigate this relevant target group with a more realistic matrix and more different aromas.

## Figures and Tables

**Table 1:** Aroma concentrations in the samples and overview of what samples were included in each experiment. DA = descriptive analysis.

Included in	Aroma Type	Sucrose Concentration (% *w/w*)		
		2.5	5	7.5
DA	Banana	1.0 mL/kg	1.0 mL/kg	1.0 mL/kg
DA	Elderflower	1.0 mL/kg	1.0 mL/kg	1.0 mL/kg
DA	Honey	0.5 mL/kg	0.5 mL/kg	0.5 mL/kg
DA	Raspberry	1.0 mL/kg	1.0 mL/kg	1.0 mL/kg
DA + consumer study	Vanilla	1.0 mL/kg	1.0 mL/kg	1.0 mL/kg
Consumer study	No aroma	-	-	-

**Table 2:** Means ± standard (std.) errors and *p*-values for each design factor and attribute included in the DA. * indicates interaction. Significant effects are marked in bold. For sweet and sour taste, significant differences found with Tukey’s comparison test are indicated with letters. Samples sharing a letter in each column are not significantly different.

		Sweet Aroma Ratings	Sour Aroma Ratings	Intensity of Aroma Ratings	Sweet Taste Ratings		Sour Taste Ratings		Intensity of Flavor Ratings
Sucrose		*p* = 0.825	*p* = 0.920	*p* = 0.425	***p* < 0.0001**		***p* < 0.0001**		***p* < 0.0001**
Aroma		***p* < 0.0001**	***p* < 0.0001**	***p* < 0.0001**	***p* < 0.0001**		***p* < 0.0001**		***p* < 0.0001**
Sucrose * Aroma		*p* = 0.969	*p* = 0.488	*p* = 0.715	***p* = 0.044**		***p* = 0.007**		*p* = 0.246
2.5% sucrose	Vanilla	12.28 ± 0.61	1.48 ± 0.48	10.30 ± 0.97	8.46 ± 0.86	de	2.73 ± 0.80	ab	7.49 ± 0.88
	Honey	11.21 ± 0.69	1.66 ± 0.32	11.13 ± 0.64	7.04 ± 0.94	cd	3.21 ± 0.79	ab	7.56 ± 0.92
	Banana	10.75 ± 0.80	5.10 ± 0.86	11.35 ± 0.68	6.19 ± 0.89	bc	4.50 ± 0.80	bc	6.91 ± 0.81
	Raspberry	4.04 ± 0.62	11.67 ± 0.69	4.91 ± 0.72	2.93 ± 0.50	a	11.33 ± 0.62	e	2.56 ± 0.44
	Elderflower	2.38 ± 0.36	12.43 ± 0.44	3.61 ± 0.55	2.31 ± 0.43	a	10.65 ± 0.81	de	1.99 ± 0.33
5.0% sucrose	Vanilla	12.58 ± 0.52	1.34 ± 0.28	11.30 ± 0.76	11.71 ± 0.69	gh	1.39 ± 0.27	a	10.88 ± 0.71
	Honey	11.15 ± 0.72	2.60 ± 0.66	11.42 ± 0.55	11.21 ± 0.65	fgh	2.55 ± 0.60	ab	10.25 ± 0.71
	Banana	10.59 ± 0.79	5.69 ± 0.86	11.33 ± 0.61	10.26 ± 0.73	efg	4.53 ± 0.75	bc	9.21 ± 0.68
	Raspberry	4.59 ± 0.71	10.56 ± 0.89	5.07 ± 0.76	5.81 ± 0.83	bc	10.22 ± 0.82	de	3.74 ± 0.51
	Elderflower	2.57 ± 0.51	12.42 ± 0.43	4.17 ± 0.72	5.10 ± 0.69	b	8.92 ± 0.90	d	3.57 ± 0.55
7.5% sucrose	Vanilla	12.59 ± 0.63	1.80 ± 0.55	11.46 ± 0.80	13.88 ± 0.29	i	1.12 ± 0.26	a	12.73 ± 0.38
	Honey	11.58 ± 0.58	1.84 ± 0.45	10.95 ± 0.64	13.10 ± 0.20	hi	1.88 ± 0.37	a	12.00 ± 0.45
	Banana	10.61 ± 0.83	5.60 ± 0.91	10.90 ± 0.66	12.86 ± 0.43	hi	4.67 ± 0.82	bc	11.24 ± 0.60
	Raspberry	3.99 ± 0.60	10.72 ± 0.71	5.46 ± 0.73	9.38 ± 0.79	ef	8.99 ± 0.88	d	6.98 ± 0.72
	Elderflower	2.17 ± 0.40	12.05 ± 0.62	3.16 ± 0.62	9.42 ± 0.81	ef	6.59 ± 0.96	c	5.32 ± 0.71

**Table 3:** Means ± std. errors for the ratings of sweet aroma and sweet taste intensity for males and females, respectively, as well as *p*-values for the factors in the models. Significant effects are marked in bold.

		Males		Females	
		Sweet Aroma Ratings	Sweet Taste Ratings	Sweet Aroma Ratings	Sweet Taste Ratings
Age		*p* = 0.530	*p* = 0.996	*p* = 0.244	***p* = 0.009**
Sucrose		***p* = 0.012**	***p* < 0.001**	*p* = 0.871	***p* < 0.001**
Aroma		***p* < 0.001**	***p* = 0.004**	***p* < 0.001**	***p* < 0.001**
Age*sucrose		*p* = 0.117	*p* = 0.378	*p* = 0.372	*p* = 0.869
Age*aroma		*p* = 0.213	***p* = 0.021**	*p* = 0.566	*p* = 0.472
Sucrose*aroma		***p* = 0.010**	***p* = 0.001**	*p* = 0.811	***p* < 0.001**
2.5% sucrose	No aroma	1.3 ± 0.1	2.9 ± 0.3	1.5 ± 0.1	3.1 ± 0.2
	Vanilla	6.2 ± 0.3	4.4 ± 0.3	7.2 ± 0.2	5.4 ± 0.2
5.0% sucrose	No aroma	1.4 ± 0.1	5.9 ± 0.2	1.6 ± 0.2	6.6 ± 0.2
	Vanilla	6.8 ± 0.3	6.1 ± 0.2	7.2 ± 0.2	6.7 ± 0.2
7.5% sucrose	No aroma	1.4 ± 0.1	7.0 ± 0.3	1.5 ± 0.1	7.2 ± 0.2
	Vanilla	5.6 ± 0.3	7.5 ± 0.2	7.2 ± 0.2	8.4 ± 0.1

**Table 4:** Cluster liking centroids for each sample together with the demographics for each cluster. Numbers in brackets shows the percentages from the total number of people, males, and 19–21-year-olds, respectively.

Liking Clusters		1 Sweet Likers	2 Neutrals	3 Sweet Dislikers
2.5% sucrose	No aroma	3.00	5.38	4.00
	Vanilla	3.00	4.98	2.68
5.0% sucrose	No aroma	4.48	5.40	3.09
	Vanilla	4.62	5.73	2.64
7.5% sucrose	No aroma	5.57	5.40	2.51
	Vanilla	5.43	5.80	2.13
Total number of people (%)		21 (16.3)	55 (42.6)	53 (41.1)
Number of males (%)		14 (27.5)	28 (54.9)	9 (17.6)
Number of 19-21-year-olds (%)		11 (19.6)	25 (44.6)	20 (35.7)

**Table 5:** Means ± std. errors for the ratings of sweet aroma and sweet taste intensity as well as *p*-values for the factors in the models. Significant effects are marked in bold.

		Sweet Aroma Ratings	Sweet Taste Ratings
Cluster		*p* = 0.542	***p* = 0.001**
Sucrose		*p* = 0.212	***p* < 0.001**
Aroma		***p* < 0.001**	***p* < 0.001**
Cluster*sucrose		*p* = 0.307	*p* = 0.864
Cluster*aroma		*p* = 0.959	*p* = 0.783
Sucrose*aroma		*p* = 0.303	***p* < 0.001**
2.5% sucrose	No aroma	1.4 ± 0.1	3.0 ± 0.2
	Vanilla	6.8 ± 0.2	5.0 ± 0.2
5.0% sucrose	No aroma	1.5 ± 0.1	6.3 ± 0.2
	Vanilla	7.0 ± 0.2	6.5 ± 0.2
7.5% sucrose	No aroma	1.4 ± 0.1	7.1 ± 0.2
	Vanilla	6.6 ± 0.2	8.0 ± 0.1

## References

[1] Johnson R.K., Appel L.J., Brands M., Howard B.V., Lefevre M., Lustig R.H., Sacks F., Steffen L.M., Wylie-Rosett J. (2009). Dietary sugars intake and cardiovascular health a scientific statement from the American heart association. Circulation.

[2] Hu F.B. (2013). Resolved: There is sufficient scientific evidence that decreasing sugar-sweetened beverage consumption will reduce the prevalence of obesity and obesity-related diseases. Obes. Rev..

[3] DuBois G.E., Prakash I. (2012). Non-Caloric Sweeteners, Sweetness Modulators, and Sweetener Enhancers. Annu. Rev. Food Sci. Technol..

[4] Markey O., Lovegrove J.A., Methven L. (2015). Sensory profiles and consumer acceptability of a range of sugar-reduced products on the UK market. Food Res. Int..

[5] Labbe D., Damevin L., Vaccher C., Morgenegg C., Martin N. (2006). Modulation of perceived taste by olfaction in familiar and unfamiliar beverages. Food Qual. Prefer..

[6] Stieger M., Velde F. (2013). van de Microstructure, texture and oral processing: New ways to reduce sugar and salt in foods. Curr. Opin. Colloid Interface Sci..

[7] Alcaire F., Antúnez L., Vidal L., Giménez A., Ares G. (2017). Aroma-related cross-modal interactions for sugar reduction in milk desserts: Influence on consumer perception. Food Res. Int..

[8] Hutchings S.C., Low J.Y.Q., Keast R.S.J. (2019). Sugar reduction without compromising sensory perception. An impossible dream?. Crit. Rev. Food Sci. Nutr..

[9] Labbe D., Rytz A., Morgenegg C., Ali S., Martin N. (2007). Subthreshold olfactory stimulation can enhance sweetness. Chem. Senses.

[10] Labbe D., Martin N. (2009). Impact of novel olfactory stimuli at supra and subthreshold concentrations on the perceived sweetness of sucrose after associative learning. Chem. Senses.

[11] Tournier C., Sulmont-Rossé C., Sémon E., Vignon A., Issanchou S., Guichard E. (2009). A study on texture-taste-aroma interactions: Physico-chemical and cognitive mechanisms. Int. Dairy J..

[12] Burseg K.M.M., Camacho S., Knoop J., Bult J.H.F. (2010). Sweet taste intensity is enhanced by temporal fluctuation of aroma and taste, and depends on phase shift. Physiol. Behav..

[13] Boakes R.A., Hemberger H. (2012). Odour-modulation of taste ratings by chefs. Food Qual. Prefer..

[14] Wang G., Hayes J., Ziegler G., Roberts R., Hopfer H. (2018). Dose-Response Relationships for Vanilla Flavor and Sucrose in Skim Milk: Evidence of Synergy. Beverages.

[15] Wang G., Bakke A.J., Hayes J.E., Hopfer H. (2019). Demonstrating cross-modal enhancement in a real food with a modified ABX test. Food Qual. Prefer..

[16] Schifferstein H.N.J., Verlegh P.W.J. (1996). The role of congruency and pleasantness in odor-induced taste enhancement. Acta Psychol..

[17] Djordjevic J., Zatorre R.J., Jones-Gotman M. (2004). Odor-induced changes in taste perception. Exp. Brain Res..

[18] Oliveira D., Antúnez L., Giménez A., Castura J.C., Deliza R., Ares G. (2015). Sugar reduction in probiotic chocolate-flavored milk: Impact on dynamic sensory profile and liking. Food Res. Int..

[19] Charles M., Endrizzi I., Aprea E., Zambanini J., Betta E., Gasperi F. (2017). Dynamic and static sensory methods to study the role of aroma on taste and texture: A multisensory approach to apple perception. Food Qual. Prefer..

[20] Cliff M., Noble A.C. (1990). Time-Intensity Evaluation of Sweetness and Fruitiness and Their Interaction in a Model Solution. J. Food Sci..

[21] Stevenson R.J., Prescott J., Boakes R.A. (1999). Confusing tastes and smells: How odours can influence the perception of sweet and sour tastes. Chem. Senses.

[22] Frank R.A., Byram J. (1988). Taste-smell interactions are tastant and odorant dependent. Chem. Senses.

[23] Barba C., Beno N., Guichard E., Thomas-Danguin T. (2018). Selecting odorant compounds to enhance sweet flavor perception by gas chromatography/olfactometry-associated taste (GC/O-AT). Food Chem..

[24] Auvray M., Spence C. (2008). The multisensory perception of flavour. Conscious. Cogn..

[25] Prescott J. (2012). Chemosensory learning and flavour: Perception, preference and intake. Physiol. Behav..

[26] Doty R.L., Cameron E.L. (2009). Sex differences and reproductive hormone influences on human odor perception. Physiol. Behav..

[27] Cooper R.M., Bilash I., Zubek J.P. (1959). The effect of age on taste sensitivity. J. Gerontol..

[28] Moore L.M., Nielsen C.R., Mistretta C.M. (1982). Sucrose taste thresholds: Age-related differences. Journals Gerontol..

[29] Mojet J., Christ-Hazelhof E., Heidema J. (2001). Taste Perception with Age: Generic or Specific Losses in Threshold Sensitivity to the Five Basic Tastes?. Chem. Senses.

[30] Forde C.G., Delahunty C.M. (2004). Understanding the role cross-modal sensory interactions play in food acceptability in younger and older consumers. Food Qual. Prefer..

[31] Yeomans M.R., Tepper B.J., Rietzschel J., Prescott J. (2007). Human hedonic responses to sweetness: Role of taste genetics and anatomy. Physiol. Behav..

[32] Deglaire A., Méjean C., Castetbon K., Kesse-Guyot E., Hercberg S., Schlich P. (2015). Associations between weight status and liking scores for sweet, salt and fat according to the gender in adults (The Nutrinet-Santé study). Eur. J. Clin. Nutr..

[33] Mahar A., Duizer L.M. (2007). The effect of frequency of consumption of artificial sweeteners on sweetness liking by women. J. Food Sci..

[34] Garneau N.L., Nuessle T.M., Mendelsberg B.J., Shepard S., Tucker R.M. (2018). Sweet liker status in children and adults: Consequences for beverage intake in adults. Food Qual. Prefer..

[35] Proserpio C., Laureati M., Invitti C., Cattaneo C., Pagliarini E. (2017). BMI and gender related differences in cross-modal interaction and liking of sensory stimuli. Food Qual. Prefer..

[36] Philipsen D.H., Clydesdale F.M., Griffin R.W., Stern P. (1995). Consumer Age Affects Response to Sensory Characteristics of a Cherry Flavored Beverage. J. Food Sci..

[37] Lavin J.G., Lawless H.T. (1998). Effects of color and odor on judgments of sweetness among children and adults. Food Qual. Prefer..

[38] Storey M.L., Forshee R.A., Anderson P.A. (2006). Beverage consumption in the US population. J. Am. Diet. Assoc..

[39] Ervin R.B., Ogden C.L. (2013). Consumption of added sugars among U.S. adults, 2005–2010. NCHS Data Brief.

[40] Pedersen A.N., Christensen T., Matthiessen J., Knudsen V.K., Rosenlund-Sørensen M., Biltoft-Jensen A., Hinsch H.-J., Ygil K.H., Kørup K., Saxholt E. (2015). Dietary habits in Denmark 2011–2013. Main results.

[41] Mendy V.L., Vargas R., Payton M., Cannon-Smith G. (2017). Association between Consumption of Sugar-Sweetened Beverages and Sociodemographic Characteristics among Mississippi Adults. Prev. Chronic Dis..

[42] Poinot P., Arvisenet G., Ledauphin J., Gaillard J.L., Prost C. (2013). How can aroma-related cross-modal interactions be analysed? A review of current methodologies. Food Qual. Prefer..

[43] Zheng M., Rangan A., Olsen N.J., Andersen L.B., Wedderkopp N., Kristensen P., Grøntved A., Ried-Larsen M., Lempert S.M., Allman-Farinelli M. (2015). Substituting sugar-sweetened beverages with water or milk is inversely associated with body fatness development from childhood to adolescence. Nutrition.

[44] Williams E.J. (1949). Experimental Designs Balanced for the Estimation of Residual Effects of Treatments. Aust. J. Chem..

[45] Addinsoft XLSTAT Statistical and Data Analysis Solution. https://www.xlstat.com.

[46] Chifala W.M., Polzella D.J. (1995). Smell and taste classification of the same stimuli. J. Gen. Psychol..

[47] Stevenson R.J., Prescott J., Boakes R.A. (1995). The acquisition of taste properties by odors. Learn. Motiv..

[48] Stevenson R.J., Boakes R.A., Prescott J. (1998). Changes in Odor Sweetness Resulting from Implicit Learning of a Simultaneous Odor-Sweetness Association: An Example of Learned Synesthesia. Learn. Motiv..

[49] Keast R.S.J., Breslin P.A.S. (2002). An overview of binary taste–taste interactions. Food Qual. Prefer..

[50] Clark C.C., Lawless H.T. (1994). Limiting response alternatives in time-intensity scaling: An examination of the halo-dumping effect. Chem. Senses.

[51] Sakai N., Kobayakawa T., Gotow N., Saito S., Imada S. (2001). Enhancement of Sweetness Ratings of Aspartame by a Vanilla Odor Presented Either by Orthonasal or Retronasal Routes. Percept. Mot. Skills.

[52] Uddannelses- og, Forskningsministeriet Optag 2017—Alder (Enrolment 2017—Age). https://ufm.dk/uddannelse/statistik-og-analyser/sogning-og-optag-pa-videregaende-uddannelser/grundtal-om-sogning-og-optag/ansogere-og-optagne-fordelt-pa-kon-alder-og-adgangsgrundlag.

[53] Hyde R.J., Feller R.P. (1981). Age and sex effects on taste of sucrose, NaCl, citric acid and caffeine. Neurobiol. Aging.

[54] Weiffenbach J.M., Baum B.J., Burghauser R. (1982). Taste thresholds: Quality specific variation with human aging. J. Gerontol..

[55] Yamauchi Y., Endo S., Yoshimura I. (2002). A new whole-mouth gustatory test procedure: II. Effects of aging, gender and smoking. Acta Otolaryngol..

[56] Mojet J., Christ-Hazelhof E., Heidema J. (2005). Taste perception with age: Pleasantness and its relationships with threshold sensitivity and supra-threshold intensity of five taste qualities. Food Qual. Prefer..

[57] Dinnella C., Monteleone E., Piochi M., Spinelli S., Prescott J., Pierguidi L., Gasperi F., Laureati M., Pagliarini E., Predieri S. (2018). Individual variation in PROP status, fungiform papillae density, and responsiveness to taste stimuli in a large population sample. Chem. Senses.

[58] Siefarth C., Tyapkova O., Beauchamp J., Schweiggert U., Buettner A., Bader S. (2011). Influence of polyols and bulking agents on flavour release from low-viscosity solutions. Food Chem..

[59] Iatridi V., Hayes J.E., Yeomans M.R. (2019). Reconsidering the classification of sweet taste liker phenotypes: A methodological review. Food Qual. Prefer..

[60] Tuorila H., Keskitalo-Vuokko K., Perola M., Spector T., Kaprio J. (2017). Affective responses to sweet products and sweet solution in British and Finnish adults. Food Qual. Prefer..

[61] Yang Q., Kraft M., Shen Y., MacFie H., Ford R. (2019). Sweet Liking Status and PROP Taster Status impact emotional response to sweetened beverage. Food Qual. Prefer..

[62] Baker A.H., Wardle J. (2003). Sex differences in fruit and vegetable intake in older adults. Appetite.

[63] Rappoport L., Peters G.R., Downey R., McCann T., Huff-Corzine L. (1993). Gender and Age Differences in Food Cognition. Appetite.

[64] Roininen K., Tuorila H., Zandstra E.H., De Graaf C., Vehkalahti K., Stubenitsky K., Mela D.J. (2001). Differences in health and taste attitudes and reported behaviour among finnish, Dutch and British consumers: A cross-national validation of the health and taste attitude scales (HTAS). Appetite.

[65] Wardle J., Haase A.M., Steptoe A., Nillapun M., Jonwutiwes K., Bellisle F. (2004). Gender Differences in Food Choice: The Contribution of Health Beliefs and Dieting. Ann. Behav. Med..

[66] Peterson J.M., Bartoshuk L.M., Duffy V.B. (1999). Intensity and Preference for Sweetness is Influenced by Genetic Taste Variation. J. Am. Diet. Assoc..

[67] Methven L., Xiao C., Cai M., Prescott J. (2016). Rejection thresholds (RjT) of sweet likers and dislikers. Food Qual. Prefer..

[68] Looy H., Callaghan S., Weingarten H.P. (1992). Hedonic response of sucrose likers and dislikers to other gustatory stimuli. Physiol. Behav..

[69] Kim J.-Y., Prescott J., Kim K.-O. (2014). Patterns of sweet liking in sucrose solutions and beverages. Food Qual. Prefer..

[70] Amsellem S., Ohla K. (2016). Perceived odor-taste congruence influences intensity and pleasantness differently. Chem. Senses.

